# Use of eHealth Platforms and Apps to Support Monitoring and Management of Home-Quarantined Patients With COVID-19 in the Province of Trento, Italy: App Development and Implementation

**DOI:** 10.2196/25713

**Published:** 2021-05-31

**Authors:** Lorenzo Gios, Giulia Crema Falceri, Stefano Micocci, Luigi Patil, Sara Testa, Simona Sforzin, Ettore Turra, Diego Conforti, Giulia Malfatti, Monica Moz, Andrea Nicolini, Paolo Guarda, Alessandro Bacchiega, Carlo Mion, Michele Marchesoni, Rosa Maimone, Pietro Benedetto Molini, Alberto Zanella, Venet Osmani, Oscar Mayora-Ibarra, Stefano Forti

**Affiliations:** 1 TrentinoSalute4.0 – Competence Center for Digital Health of the Province of Trento Trento Italy; 2 Azienda Provinciale per i Servizi Sanitari (APSS) Trento Italy; 3 Fondazione Bruno Kessler Trento Italy; 4 Provincia Autonoma di Trento Trento Italy; 5 Facoltà di Giurisprudenza, Università degli Studi di Trento Trento Italy

**Keywords:** telemedicine, telemonitoring, quarantine management, COVID-19, connected care

## Abstract

**Background:**

Italy was the first country to largely experience the COVID-19 epidemic among other Western countries during the so-called first wave of the COVID-19 pandemic. Proper management of an increasing number of home-quarantined individuals created a significant challenge for health care authorities and professionals. This was especially true when considering the importance of remote surveillance to detect signs of disease progression and consequently regulate access to hospitals and intensive care units on a priority basis.

**Objective:**

In this paper, we report on an initiative promoted to cope with the first wave of the COVID-19 epidemic in the Spring/Summer of 2020, in the Autonomous Province of Trento, Italy. A purposefully built app named TreCovid19 was designed to provide dedicated health care staff with a ready-to-use tool for remotely monitoring patients with progressive symptoms of COVID-19, who were home-quarantined during the first wave of the epidemic, and to focus on those patients who, based on their self-reported clinical data, required a quick response from health care professionals.

**Methods:**

TreCovid19 was rapidly developed to facilitate the monitoring of a selected number of home-quarantined patients with COVID-19 during the very first epidemic wave. The app was built on top of an existing eHealth platform, already in use by the local health authority to provide home care, with the following functionalities: (1) to securely collect and link demographic and clinical information related to the patients and (2) to provide a two-way communication between a multidisciplinary health care team and home-quarantined patients. The system supported patients to self-assess their condition and update the multidisciplinary team on their health status. The system was used between March and June 2020 in the province of Trento.

**Results:**

A dedicated multidisciplinary group of health care professionals adopted the platform over a period of approximately 3 months (from March-end to June 2020) to monitor a total of 170 patients with confirmed COVID-19 during home quarantine. All patients used the system until the end of the initiative. The TreCovid19 system has provided useful insights of possible viability and impact of a technological–organizational asset to manage a potentially critical workload for the health care staff involved in the periodic monitoring of a relevant number of quarantined patients, notwithstanding its limitations given the rapid implementation of the whole initiative.

**Conclusions:**

The technological and organizational model adopted in response to the COVID-19 pandemic was developed and finalized in a relatively short period during the initial few weeks of the epidemic. The system successfully supported the health care staff involved in the periodic monitoring of an increasing number of home-quarantined patients and provided valuable data in terms of disease surveillance.

## Introduction

Since the first cases were reported in December 2019, the COVID-19 outbreak caused by the novel coronavirus SARS-CoV-2 has spread rapidly, creating an unprecedented challenge to health care systems worldwide [1].

The COVID-19 pandemic has considerably altered health care systems worldwide and put at risk their sustainability, while boosting the adoption of telemedicine, which has been designed to address the challenges related to patients with COVID-19 [2]. During the pandemic, medical institutions were rapidly facing a massive tsunami of patients requiring hospital treatment, with critical consequences in terms of health care staff workload and dwindling of medical care resources. To prevent the collapse of the global health care system, many countries have advocated for infected patients with mild symptoms to stay at home and self-quarantine [3]. However, it has been observed that the condition of some home-quarantined patients became severe or critical as the disease progressed [4], leading to a delay in timely treatment and hospitalization of these patients and, consequently, rapid deterioration or even death.

Italy was the first country to largely experience the COVID-19 epidemic among other Western countries, and the first country in Europe to impose a general lockdown in March 2020 so as to limit the spread of COVID-19 [5]. From an epidemiological perspective, the COVID-19 epidemic had spread at varying levels across the different regions of Italy with a significant geographic heterogeneity in terms of the number of cases and dynamics of the outbreak [6]. The first cases (n=4) officially reported in the Autonomous Province of Trento (PAT) were identified on March 3, 2020, whereas by the end of March, the number of individuals infected with COVID-19 increased to 2529. In terms of individuals infected per day, the peak of the first wave was reached on March 21, 2020, with 239 newly infected patients, whereas the highest number of daily deaths (n=18) was reached on March 30, 2020. The massive scale-up of infected patients exposed the provincial health care system to an urgent, wide, and rapid organizational and logistic rearrangement during the course of the very first epidemic wave.

In fact, within 4 weeks of the very first cases reported, an exponentially increasing number of patients needed monitoring, active support, and prompt hospitalization, and a significant proportion of patients required intensive care. The peak of the burden for health care services was reached on April 8, 2020, with 311 patients hospitalized in an infectious diseases department, 77 patients managed in intensive care units, and 43 patients managed in high-intensity units. Only 3 months from the very onset of the pandemic, June 2, 2020, was the first day with 0 COVID-19 cases reported in the province of Trento, whereas the last infected patient was discharged from the intensive care unit on June 12, 2020.

The COVID-19 pandemic had suddenly exposed deficiencies in the whole health care system, revealing high uncertainty on key issues such as increasing difficulty in tracing the exact transmission route of the virus, inadequacy of the diagnostic testing system, and the lack of clear monitoring procedures in the case of home-quarantined patients and therapeutic approaches [7]. Ensuring proper management of an increasing number of home-quarantined individuals posed a demanding challenge to the health care authorities and professionals, considering the key role of strict monitoring in order to detect disease aggravation in view of prompt hospitalization and regulating access to hospitals and intensive care units only when needed.

Within this critical and rapidly changing scenario, there was a scattered phenomenon of swift and spontaneous—albeit valuable—attempts to adopt digital tools to support the health care system in dealing with this unexpected epidemic, particularly in the field of remote monitoring. Although some forms of remote monitoring of home-quarantined patients have been implemented in other countries during the very first stage of the pandemic [8,9], no such initiatives were undertaken in Italy. At the same time, the COVID-19 pandemic has been considered as a missed opportunity to improve telemedicine [6,10], which is still a scattered and embryonic phenomenon at the national level.

In this paper, we report on the initiative designed and implemented to cope with the first wave of the COVID-19 epidemic in Spring/Summer 2020 in the province of Trento, Italy. In the very first weeks after the outbreak, a telemedicine tool was purposefully developed to provide home-quarantined patients with COVID-19 with an app named TreCovid19. The app was linked to an already existing telemedicine system that was in use by nurses who provided home care. The app and the platform were set up within an extremely short period to enable an automated monitoring system supporting health care staff when dealing with an ever-increasing number of infected patients. This was achieved by merging organizational and technological components, that is, by embedding a telehealth service and related activities into the framework of health care procedures put in place to face the epidemic’s impact, particularly in terms of monitoring of home-quarantined patients.

The TreCovid19 tool was developed within an initiative promoted and coordinated by the Competence Centre on Digital Health of PAT, TrentinoSalute4.0 (TS4.0) [11].

## Methods

### Contextual Factors: Collaboration Among Health Care Bodies in Trentino

A key component of the initiative described in the present case study lies in a specific contextual factor characterizing the collaboration between health care bodies and policy and research stakeholders in the province of Trento. TS4.0 was formally established in 2016 with an Act of the local government as a partnership among three relevant stakeholders in Trentino: the Department of Health and Social Policies of PAT, the local Healthcare Trust of the Autonomous Province of Trento (Azienda Provinciale per i Servizi Sanitari [APSS]), and the Bruno Kessler Foundation (FBK)—a research entity with particular focus on the applicative dimension of technology in the field of digital health. This alliance has been established for strengthening cooperation among the three institutions and coordinating the eHealth agenda in Trentino; in May 2020, TS4.0 officially became a Joint Research Unit. Specific financial funding has been allocated by the province of Trento to support TS4.0 coordination activities, whereas a specific TS4.0 Steering Committee has been formed comprising representatives of the partner institutions (ie, PAT, APSS, and FBK). The Steering Committee is in charge of defining and approving the overall strategy of TS4.0 and prioritizing areas of action, as well as monitoring a smooth implementation of related activities. In recent years, this pre-existing framework of collaboration has provided a solid basis to a number of initiatives in the field of digital health (from piloting to the delivery of eHealth services), including telemedicine projects [12].

### Organizational Response

In view of the first epidemic wave in the province of Trento, health care authorities set up a COVID-19 Special Unit, established to deliver a general strategy to cope with the pandemic and to ensure proper management of positive cases, from monitoring to hospitalization and treatment. This unit was strongly linked with PAT (in particular, with the Department of Health and Social Policies), as well as with the Directorate General of APSS, in order to ensure proper decision-making. The COVID-19 Special Unit was also linked with ad hoc contact points within different public health institutions at the local level (eg, hospitals and local districts). Patients with a probable or confirmed COVID-19 infection were reported to this unit through different channels, namely, the provincial registry of citizens with positive swabs, the prevention department, and via general practitioners (GPs).

The monitoring of home-quarantined patients with COVID-19 was considered a key component in the management of the epidemic, particularly in the first phase of the outbreak. This was despite the fact that it represented a challenging action considering the relative uncertainty about clinical manifestations and related indicators in the early stage of the epidemic [7]. Therefore, within the COVID-19 Special Unit, a selected group of health care professionals were put in charge of monitoring patients at the provincial level, namely, 2 medical doctors and 2 nurse coordinators were in charge of managing and coordinating the monitoring activities and 13 nurses, 2 medical doctors (specialists), and 1 medical doctor from the Special Continuity Care Unit (so-called Unità Speciali di Continuità Assistenziale [USCA]) were in charge of performing the actual monitoring of COVID-19–positive cases. A total of nearly 80 health care professionals were also involved in the monitoring phase at the community level. The monitoring of home-quarantined patients was initially set up using the following procedure: medical doctors and nurses from the COVID-19 Special Unit phoned the home-quarantined patients twice a day (morning and afternoon) to collect information about their clinical status and progression of disease symptoms (if any). Data collected included the patients’ self-reported body temperature, perceived pain, level of fatigue, dyspnoea, level of consciousness, and presence of deep vein thrombosis. This continuous and remote monitoring performed through periodical phone calls was set up to detect the progression of disease and to support direct referral to the GP for clinical assessment and access to the emergency room for evaluation with consequent hospitalization, if necessary.

Within a few weeks since the onset of the COVID-19 outbreak in the province, the rapidly increasing number of home-quarantined patients who needed monitoring created a demanding challenge among the health care authorities and professionals.

### Selecting a Priority During the Very Start of the COVID-19 Outbreak

Since the onset of the COVID-19 outbreak in the province of Trento, TS4.0—as the leading player in the field of digital health within the province—was immediately given the mandate to facilitate the process of designing and delivering eHealth services to support a swift reaction to the pandemic. No specific extra-financial funding was acquired for this task to be implemented during the emergency period, as this has been considered part of the standard coordination activities of TS4.0.

Within this context, the TS4.0 Steering Committee identified the urgent need for core actions such as the rapid implementation of a digital solution to support health care staff in charge of monitoring patients with COVID-19, particularly those who are home-quarantined. This decision was the result of both internal consultations and a prompt negotiation with the COVID-19 Special Unit (the overview of the organizational asset is provided in Multimedia Appendix 1). The reasons behind this decision were both organizational and technological. From an organizational viewpoint, it was rapidly clear that the number of home-quarantined citizens was steadily increasing in the very first weeks since the onset of the epidemic and health care staff in charge of monitoring these patients were extremely exposed to overwork. Furthermore, because clinical management of the patients in the hospital was clearly a sole responsibility of the health care staff, the periodical collection of information on home-quarantined patients could be (partially) assigned to the patients themselves, by and with reliable and ready-to-use self-care or self-monitoring solutions. From a technological viewpoint, it was considered that specific apps for telemonitoring and self-care of patients had already been piloted and used in the context of previous projects, and a technological tool in use for the management of integrated home care in Trentino was already available as part of the standard care (the so-called “@home platform”).

In terms of technological assets, a multidisciplinary approach was also adopted. A working group comprising medical doctors, nurses, information technology (IT) specialists, technologists, researchers, and project managers from the PAT, APSS, and FBK was set up under the coordination of TS4.0, with the aim of collecting clinical requirements and designing, testing, and implementing technological tools.

As previously mentioned, the project was based on an already existing partnership among the Department of Health and Social Policies of PAT, APSS, and FBK. This previous collaboration represented the ideal basis to quickly develop and deliver a ready-to-use tool for supporting health care staff in charge of monitoring patients. Clinical colleagues from APSS, including members of the COVID-19 Special Unit, were responsible for setting up the clinical assumptions and criteria behind the technological asset, whereas IT professionals from APSS and FBK were in charge of developing the app and linking the system with the already-existing telemedicine system, which was in use by the nurses who provided home care. PAT colleagues provided guidance and inputs in line with the provincial strategy implemented during the COVID-19 emergency period, particularly the strategy related to patient management.

### Development of an App for Monitoring Home-Quarantined Patients With COVID-19

#### Overview

The aim of rapidly incorporating a technological and organizational approach toward COVID-19 monitoring in Trentino was motivated by the need to support the health care staff in dealing with the sudden increase in the workload presented during the first stage of disease transmission.

To reach this objective, the TreCovid19 system was specifically set up to (1) regularly and automatically collect self-reported symptoms from home-quarantined patients with COVID-19 through a smartphone app; (2) translate subjective self-reports of these symptoms into numerical scales; and (3) allow a set of alerts based on specific cut-offs, periodically informing health care staff about the status of the patients and optimizing interventions and direct contacts if required.

The mobile app was embedded into an already existing telemedicine platform, adopted by home care practitioners. The core approach adopted for this endeavor was to merge organizational and technological components by embedding a telehealth service (mainly supported by a dedicated app) into the already-existing framework of clinical procedures for monitoring of home-quarantined patients.

The system was rapidly developed by designing and delivering two components: (1) an app for patients to support daily self-collection of symptoms and (2) a dashboard for medical doctors and nurses in charge of monitoring patients.

#### App to Support Patients’ Daily Self-Collection of Symptoms

**Figure 1:**
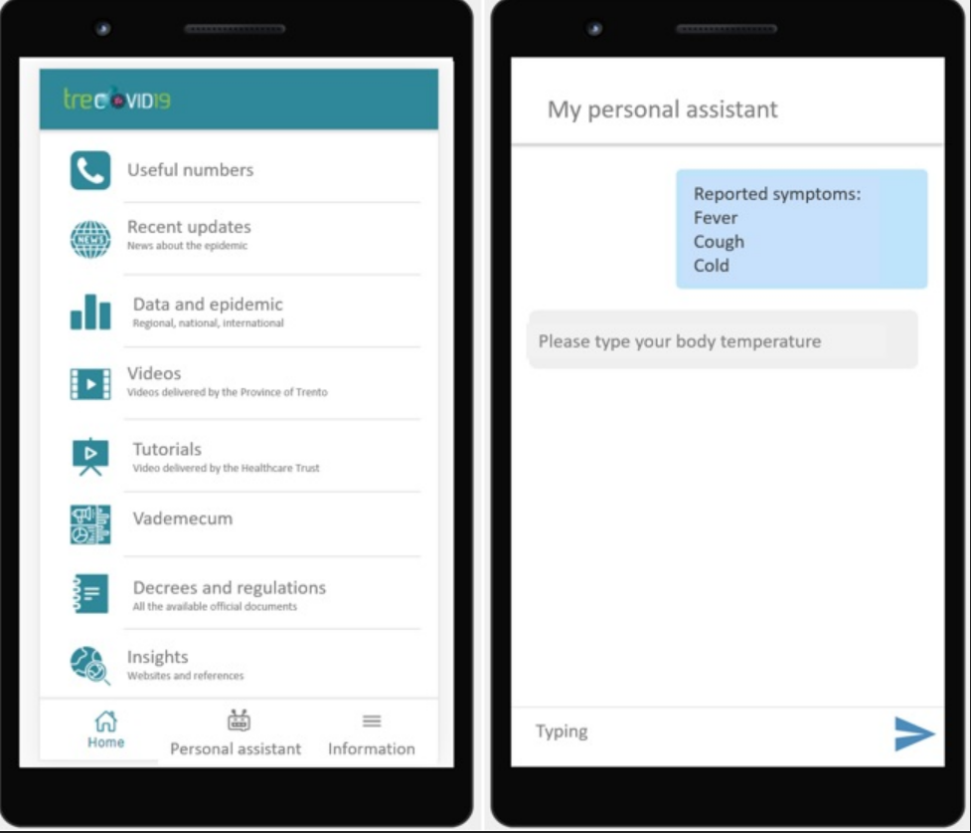
Layout of the TreCovid19 app and related functionalities.

The TreCovid19 app homepage has a number of functionalities that are divided into two main types (see Figure 1). The first category is related to providing official information about the COVID-19 pandemic and general information to cope with it, such as recent updates about the epidemic data, video tutorials delivered by APSS, tips and advice on specific safety procedures, information on regional and national decrees, and regulations related to the COVID-19 pandemic.

The second category constituted the core tool for patients and is represented by an automated chatbot functionality. The app would periodically activate a specific chat with the patient, administering a set of items to gather self-reported data on their health status and related symptoms. The different pieces of information were collected twice a day (once in the morning and once in the afternoon) and communicated in real time to the central system. In case the level of self-reported symptoms exceeded the cut-offs set by the health care staff (see Table 1), a specific alarm was sent via email to the COVID-19 Special Unit for immediate (re)action.

#### Dashboard for Health Care Staff

The dashboard for the health care staff was based on the so-called @home system [13,14]. Such a system is constructed on top of the APSS technological tool currently in use for the management of integrated home care in Trentino. The @home application was successfully adopted to improve efficiency in managing the reporting process of all interventions performed by home care case managers. The adoption of the system reduced the workload for reports management, as well as reduced paperwork for nurses.

The dashboard for the health care staff was developed using a customer relationship management commercial tool, whereas nurses could use a dedicated tool during home visits. The dashboard uses a specific Platform as a Service (PaaS), which makes it possible to operate on isolated virtual environments.

The integration currently available in the @home platform allows a proper link with consent and privacy documentations available within the official document repository, in addition to a secure link and integration with the health register, the register of operators or health care staff, and the APSS notification system.

### Setting up Clinical Requirements

To set up the COVID-19 quarantine monitoring tool (mobile app) for patients, a rapid review of the available scientific literature, technical documents, and reports was performed by the clinicians collaborating within the working group. A number of assessment scales were also reviewed by medical experts from the COVID-19 team to identify potential items to be included in the monitoring tool, such as standard validated scales [15]. A multidisciplinary approach was adopted, by setting up a working group comprising medical doctors, nurses, IT experts, and project managers, to set up an automated monitoring tool to be used in the very first wave of the epidemic. The working group was constantly in contact with and reporting to the steering committee, to ensure an effective decision-making process. It should be underlined that the unexpectedness of the first COVID-19 outbreak and related organizational complications were detrimental to both the health care staff and IT staff in developing the system in a very short period. Other major challenges rose due to the relative uncertainty regarding clinical manifestations and related indicators of COVID-19 in the first few months of the epidemic outbreak, rendering it difficult to construct a stable and reliable list of indicators to be translated into the app functionalities.

Therefore, a pragmatic decision process was adopted by the core group of medical doctors in charge of managing the provincial COVID-19 task force. After a round of internal meetings, a core set of key indicators and related cut-offs were identified, as well as core IT functionalities. The proposal was negotiated and agreed with the Steering Committee group. In addition, a shared decision was taken to group the participants into two categories according to their clinical status, as follows:

- Red group (Acv19): patients considered to be COVID-19 positive, determined based on one of the following criteria: reporting clinically relevant symptoms; a positive swab test result; or relevant clinical parameters based on radiological examination.
- Blue group (AIOcv19): cohabitants or family members living with a patient who has tested positive for COVID-19. The blue group was specifically initiated to monitor developments in the conditions of the cohabitants, specifically to signal their potential infection by the virus.

Table 1 details the key pieces of information that have been identified by the team of medical doctors and nurses in charge of monitoring the disease progression (the detailed questionnaire is provided in Multimedia Appendix 2). An automated alarm system was designed considering the shown variables and their cut-off values.

**Table 1:** Participants’ grouping, variables, and cut-offs adopted for the TreCovid19 app.

Variables	Cut-off score or criterion	
	Red group (Acv19)	Blue group (AIOcv19)
Body temperature (°C)	≥39	≥37.5
Pain (Numerical Rating Scale)	≥4	≥4
Fatigue^a^	≥7	≥7
Peripheral oxygen saturation (SpO_2_, %)^b^	≤95	N/A^c^
Dyspnoea	≥4	≥3
Level of consciousness	Confused or coma	Confused or coma
Deep vein thrombosis	Yes	Yes
Respiratory rate (per min)	≥22	≥22
Assumption of antipyretics	N/A	Yes

^a^Fatigue was measured based on a self-reported assessment scale ranging from 0 (not at all) to 10 (very much).

^b^SpO_2_ was considered only when oximeter was available.

^c^N/A: not applicable.

### Selecting the Patients

Considering the emergency context in which the telemonitoring system has been set up and delivered, researchers, health care staff, and managers decided to adopt a conservative approach. In line with a precautionary principle, only patients with relatively stable medical conditions were contacted and provided access to the app, thereby restricting the use of the app to a limited number of eligible patients. This was decided to (1) allow proper adoption of the system and related procedures in view of a potential scale-up of the initiative and (2) guarantee a controllable margin of safety for patients in this first phase of emergency.

Therefore, patients were included based on the following inclusion criteria: diagnosed with COVID-19 and home-quarantined, residents of the province of Trento, reporting relatively stable medical conditions, ability to use a smartphone or living with a cohabitant with a smartphone, and voluntary participation. Medical doctors and nurses of the COVID-19 Special Unit were in charge of the assessment of the medical conditions, based on the experience they gained through managing the very first group of patients monitored until that time. Exclusion criteria were as follows: reporting severe medical conditions, belonging to vulnerable populations (eg, those with complex general and/or chronic health conditions), and reporting specific social and/or psychological needs (eg, anxiety management).

It should be underlined that, mainly because of emergency issues, neither the exact number of potential eligible patients nor precise clinical patterns were collected and registered. Only a limited number of patients were eligible mainly because of (1) the unexpectedness of the COVID-19 epidemic, (2) the exponential increase of the number of infected patients, and (3) the relative uncertainty about the clinical manifestations and related indicators of the infection. In addition, the app had been developed within a period of two weeks, to ensure the availability of a monitoring tool to support health care staff in the shortest possible time; therefore, the app was adopted only for a convenient sample of patients. The decision of assigning the app to a patient was based on the clinical assessment performed by the health care staff in charge of monitoring the patients, following the available guidelines and experiences gained through the very first epidemic wave. Expanding the patient sample and extending the service to patients who were nonresidents of Trentino was not considered feasible.

### Setting up the Procedural Flow

The procedure of the telemedicine system is presented below. Patients were contacted via phone by the COVID-19 Special Unit—the team in charge of monitoring COVID-19 cases. Members of the team assessed potential eligibility of patients via phone per the abovementioned criteria (see section “Selecting the Patients”).

After the eligibility for being enrolled in the project was confirmed, the participants were grouped into the two abovementioned categories, namely, Red group (Acv19, comprising patients considered to be COVID-19 positive) and Blue group (AIOcv19, comprising cohabitants or family members living with a patient with confirmed COVID-19). COVID-19–positive patients and their cohabitants were invited to access the mobile app TreCovid19. Activation of the app required patients to enter their health insurance code and fiscal code, matching with the health care platform. The app was then linked with the specific clinical profile of the patient.

Nurses and medical doctors were in charge of training and assisting patients in the process of downloading and activating the app, providing telephone support in case of issues related to installation and use. The participants underwent remote quarantine management monitoring, and the app was used to automatically prompt patients from both groups to fill in the requested information twice a day (morning and afternoon). Once an alert was received by the health care staff, the participant was contacted via phone by a trained operator (either a doctor or a nurse), to remotely assess the health status and the progression of the disease.

Two potential outcomes of the assessment were set up:

- Typing error: the alarm was triggered by incorrect data, entered by mistake. In this case, a manual correction of the parameter was performed to ensure accurate tracking and recording of data. Typing errors accounted for a very small part of the generated alarms.
- No typing error: the alarm was triggered by data correctly entered by the participants.

In the latter case, the health care staff performed a telephone-based in-depth assessment of patients’ general conditions and previous parameters, resulting in one of the following scenarios: (1) continuous use of the monitoring system based on the app; (2) intensifying remote monitoring by adding periodical phone calls to the app usage; (3) direct referral to the GP for clinical assessment, in view of the need to incorporate additional interventions (eg, pulse oximeter delivery at home, home-visit scheduling, or access to the emergency room for evaluation with consequent hospitalization if necessary). In general, and based on the telephonic in-depth assessment, health care staff considered that the validity of the self-reported data was appropriate, with few exceptions related to the potential overestimation of some symptoms.

### Ethical Issues

Dedicated information sheets and informed consent were already available for the telemedicine system in use. A specific information sheet was developed for patients when downloading and activating the TreCovid19 app.

To further improve safety and reliability of the system, a set of piloting phases were performed to ensure secure transmission of data and proper functioning of the alerting system and related cut-offs. Specific data to allow proper insights about the validity of self-reported data were not collected (eg, comparison between self-collected versus health care worker–collected data). Nevertheless, based on the information gathered through the telephone-based in-depth assessments, the validity of the data collected through the app was considered acceptable. Constant supervision of the system was ensured during the project piloting phase.

## Results

**Figure 2:**
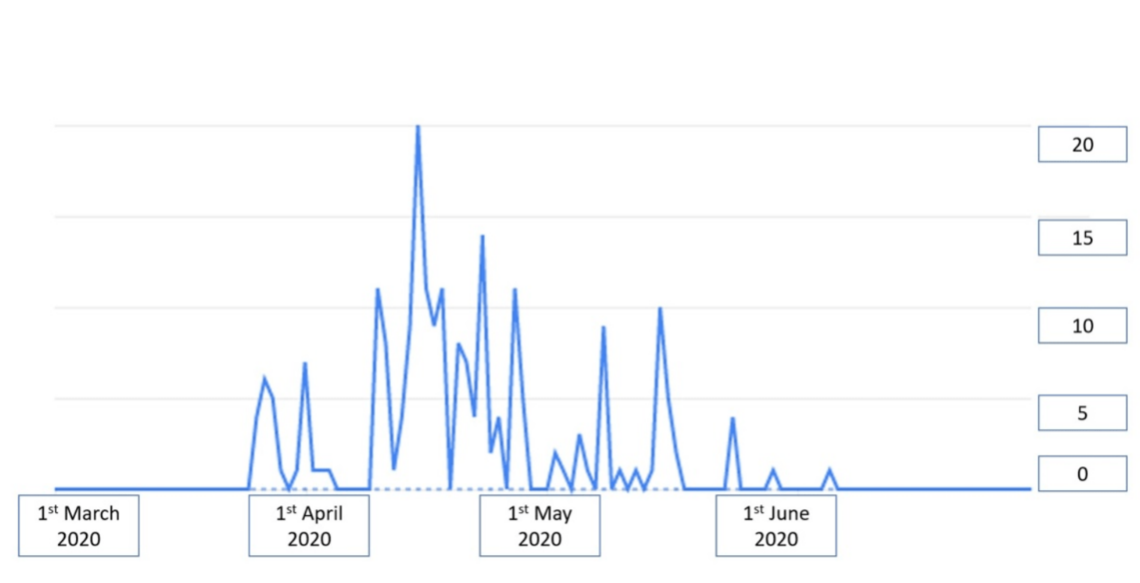
Timeline of the piloting and enrolment phases of the project.

The multidisciplinary group developed the technological platform for patient monitoring in time for its wide use during the very first months of the epidemic in the province of Trento. It should be highlighted that the first cases officially reported in the province (n=4) were identified on March 3, 2020, whereas at the end of March, the number of COVID-19 cases increased to 2529. The TreCovid19 platform was developed and made available in an extremely short period (approximately 2 weeks), which was possible owing to the existing infrastructure on which the new service had been constructed. This swift reaction enabled the monitoring of 170 home-quarantined patients with COVID-19 from March-end to June 2020 (see Figure 2). The large majority of patients monitored through the telemedicine system were enrolled in April.

Of the 170 patients specifically selected for this piloting phase, 107 (62.9%) were assigned to the red group (Acv19), that is, COVID-19–positive patients in quarantine; the remaining 63 (37.1%) were assigned to the blue group (AIOcv19), that is, cohabitants or family members living with a COVID-19–positive patient. Of the enrolled patients, half were female (85/170, 50%) with a mean age of 37.83 (SD 15.54 years). The red group sample comprised 52 (48.6%) female and 55 (51.4%) male patients (age: mean 38.95, SD 14.98 years), whereas the blue group comprised 33 (52.4%) female and 30 (47.6%) male participants (age: mean 35.92, SD 16.26 years). With regard to the red group (Acv19), 2570 monitoring measurements were collected by the app (24 measurements per user on average), whereas 1057 sets of measurements were collected by the app (17 measurements per user on average) in the blue group (AIOcv19).

On the basis of the available data, none of the patients neither deteriorated nor needed prompt hospitalization. Once recovered, the patients were simply asked to uninstall the app, which was then unlinked with the @home system as well.

## Discussion

### Principal Findings

To our knowledge, the TreCovid19 app represents a unique example of swift design and delivery of a technological innovation supporting health care staff dealing with the monitoring of home-quarantined patients during the first COVID-19 wave in Italy and in other European countries as well. The uniqueness of this experience lies in the fact that (1) the app can be considered as one of the very first telemonitoring tools launched during the first COVID-19 wave [8,9] and (2) it was launched in Italy, one of the first Western countries to be significantly hit by the COVID-19 pandemic [6]. Despite the critical contextual situation, this project has successfully achieved its goals owing to the two key strengths discussed below.

The first commendable strong point is in the presence of a pre-existing joint center for digital health at the province level. In fact, the TreCovid19 initiative was promoted and coordinated by the Competence Centre on Digital Health (TS4.0), a strategic alliance among the three core health stakeholders in the province, namely, PAT (Department of Health and Social Policies), APSS, and FBK. Despite the unexpectedness of the first COVID-19 outbreak and associated organizational complications, this collaboration represented a pivotal asset to promote swift development of the telemonitoring system within a considerably short period, promoting a prompt convergence of organizational, clinical, and technological competences within different institutions.

The second strong point is the integration of a specific telemedicine system (TreCovid19) within the health care platform already in use by thousands of citizens and an organizational asset, providing an immediately available tool for piloting a novel telemonitoring system. In fact, the pre-existing digital health infrastructures already in use for patients and the health care staff in the province of Trento [12,13,16] allowed an efficient development and delivery of a digital tool to tackle the epidemic, while the health care provincial institutions were under enormous pressure.

### Limitations

The core limitation of this initiative is related to the swift and unstable scenario in which such a telemedicine system has been developed and adopted. First, the unexpectedness of the first COVID-19 outbreak and organizational complications associated with it have exposed the health care and IT staff to a challenging scenario while developing the system within a very limited timespan.

Second, the relative uncertainty about the clinical manifestations and related indicators of COVID-19 made it difficult to construct a stable and reliable list of indicators and triggers to be translated into the app functionalities, at least in the initial months of the epidemic. Furthermore, no specific clinical, automated, or laboratory-based indicators were considered to triangulate the different pieces of self-reported information collected in the preliminary phase of this project. Because of the emergency situation and the pressing need for implementing an immediate action, the project team used a rough validation strategy based only on the completion of the requirements from the clinical team over a thorough trial analysis. For the same reason, the collection of specific data on this process was not considered critical in this phase—again, because the urgency of having a platform immediately usable for the emergency purpose was the core priority considering the increasing number of patients to be monitored. In addition, clinical evidence, as well as well-structured guidelines about specific cut-offs, were not always available when the system was designed.

This explains why researchers, health care staff, and health managers decided to adopt a conservative approach in line with a precautionary principle, and to enroll in the project a limited and convenient sample of patients with relatively stable medical conditions. This was decided to (1) allow proper evaluation of the system and related procedures and (2) guarantee a controllable margin of safety for the patients in this first phase. As a result, it was not possible to obtain a larger sample size and, therefore, to test the app and its functionalities among a larger audience, providing further validity and robustness of these findings.

The system has not been promoted as standard procedure nor scaled considering several organizational, technological, and contextual factors of emergency during the very first wave of the COVID-19 epidemic. At the same time, proper qualitative and quantitative assessments are foreseen to explore the organizational and contextual factors (eg, digital literacy, internal procedures, clinical requirements) that can potentially contribute to or hamper larger adoption of the system. Likewise, an improved co-design of the app and an update of the clinical information and related cut-offs could further improve the usability of the system. In fact, a larger implementation of a reliable telemedicine system to support patients monitoring could be particularly important during the fall/winter season of 2020 and the first part of 2021, when a relevant increase in the number of home-quarantined patients is more likely to occur.

### Lessons Learned: Do’s and Don’ts

When health care institutions are facing a *health tsunami* of sorts as the one we all experienced during the first wave of the COVID-19 pandemic, there is a clear need for a rapid reaction and swift delivery of viable procedures and tools to tackle that calamity. On the basis of this experience, we have identified some core do’s and don’ts learned through this initiative.

The first issue is related to the swift identification of the key priority to be addressed, among the different urgent issues to cope with. In our case, monitoring of home-quarantined patients with COVID-19 has been immediately considered a key component in the management of the epidemic, particularly in the first phase of the outbreak, also with a view of preventing overflowing of patients into the intensive care wards. Even if this initiative represented a challenging action considering the relative uncertainty about the clinical manifestations and related indicators in the early stage of the epidemic, developing a dedicated telemedicine system seemed to be a vital action to support the health care staff in delivering efficient health care services despite the huge increase of infected patients. This prompt identification of a list of priorities to be addressed (in this case, telemonitoring) could occur only if teamwork and well-organized collaboration among key stakeholders are already in place.

The second issue is related to the ability to select pre-existing infrastructures, adapt them in light of new contextual factors, and deliver the service within a reasonable timeframe. The provision of a telemonitoring system was essential if it was possible in the shortest timeframe possible, considering the rapid increase of the COVID-19 epidemic that was putting the health care provincial institutions under enormous pressure. Developing a new tool for monitoring COVID-19–positive patients from scratch would have resulted in an impossible and detrimental task.

In other words, the result achieved in the province of Trento could be linked to specific contextual dynamics, that we consider to be even more essential factors in the framework of sudden outbreaks and public health calamities; these include the availability of a pre-established collaboration, pre-existing technological infrastructures, and a multidisciplinary approach.

The first essential factor was the availability of a former collaboration in place among the key health organizations within the province (ie, the partnership among APSS, APT, and FBK). This was likely the key factor of success of this very initiative. In the context of a pressing pandemic, such prevailing teamwork and the availability of pre-existing tools and technological infrastructures have represented a solid foundation for coordinating a complex task such as the swift delivery of an ad hoc telemonitoring system to tackle a relatively unknown epidemic. The TS4.0 alliance has also enabled the rapid adaptation of already available eHealth platforms that have been speedily converted into a ready-to-use tool to tackle the sudden COVID-19 outbreak.

An additional factor of success was the multidisciplinary approach adopted. The harmonization between technological assets and organizational procedures, as well as putting together the clinical know-how, public health expertise, and IT knowledge, was possible only because of a multifaceted and integrative working method. The fact that medical doctors, nurses, project managers, and IT technicians were already exposed to long-term cooperation in the field of digital health resulted in a prompt and smooth cooperation within a critical context due to the COVID-19 pandemic.

Finally, we would like to further emphasize the importance of the abovementioned factors in view of potential, future scenarios similar to the COVID-19 outbreak. First, this experience has underlined the clear need for establishing solid collaboration among key health, policy, and research organizations at the local level, ideally by establishing specific joint centers for digital health. If already present, these alliances must be strengthened considering their potential pivotal role in case of tsunamis in the field of public health. In fact, this approach could guarantee the availability of an organizational asset to enable a prompt and swift reaction to emergencies. Second, promoting multidisciplinary collaboration and mutual inspiration among IT experts, public health managers, and health care staff could represent a vital long-term investment to ensure smooth convergence of different stakeholders in emergency circumstances. Lastly, the design of technological and digital health infrastructures with high levels of flexibility and adaptability could also be a strategy to pursue, to make these infrastructures flexible in case of emergency.

### Lessons Learned: Evaluation and Assessment

We discuss here a number of lessons learned through this initiative in terms of evaluation and assessment, albeit time constraints led to the lack of a robust methodological asset for evaluating and testing the system.

First, there is a clear need to develop an approach for assessing and validating technological tools developed in a strict period of time for emergency purposes, such as the one described in the present paper. Evaluating to what extent the harmonization between technological assets and organizational procedures is put in place should be a key action, even when a rapid reaction to the emergency leads to a swift adaptation and implementation of available technologies to address the containment of the health care crisis.

Second, in ideal conditions, the adoption of robust study designs should be considered, even within a context of emergency such as the COVID-19 outbreak. Setting up a clear study design (eg, case-control design) within this first phase of the project could have further improved the interpretation of outcome and results of this initiative. For instance, the idea of including a control group was discussed, but the emergency prevented the project team to design a suitable research plan, as the core objective was to deliver a ready-to-use system to support the health care staff in the very early phase of the epidemic outbreak. More advanced strategies for implementing robust study designs for reacting to emergencies should be considered in future crisis scenarios.

Third, the opportunity of a broader psychological-sociological assessment of the experiences of family members and patients monitored at home, as well as the experience of the health care staff adopting the technological tool, could have represented an added value in evaluating the initiative. Moreover, a cost-effectiveness analysis of the entire initiative would be a critical, albeit complex, task to further assess the viability and sustainability of the technological–organizational asset. 

### Conclusions

To conclude, we would like to highlight that the rapid onset of the COVID-19 pandemic urgently called upon the need for swift changes in health care provisioning, as well as the need for a rapid “design-to-deliver” approach such that specific solutions that can support the management of a rapidly increasing number of patients can be made immediately available to health care staff. Within this scenario, monitoring of home-quarantined patients with COVID-19 was a key component, as it constituted a challenging but important action to deliver efficient health care services and to control patients’ status and related hospitalization levels.

This paper describes how we managed to develop and deliver, in short time, an eHealth tool to assist health care staff in coping with an inflow of home-quarantined patients with COVID-19, even in the period of a severe and unpredictable outbreak.

The TreCovid19 system has provided useful insights of possible viability and impact of a technological–organizational asset to manage a potentially critical workload for health care staff involved in the periodic monitoring of a relevant number of quarantined patients, notwithstanding its limitations due to the rapid implementation of the whole initiative. TreCovid19 presents high potential to further support the local health care system when facing higher peaks of the COVID-19 epidemic or future health care emergencies. With this perspective, further optimization of the system, its potential extension to larger groups of home-quarantined patients in the Italian province of Trento, and a robust validation assessment of the entire model might further increase its applicability.

## Appendix

Multimedia Appendix 1 Organizational diagram depicting the structural asset and relations among the different institutions involved in the initiative.

Multimedia Appendix 2 Detailed questionnaire (Italian version with English translation) adopted for the TreCovid-19 app.

## Abbreviations

APSS: Azienda Provinciale per i Servizi Sanitari
FBK: Bruno Kessler Foundation
GP: general practitioner
IT: information technology
PaaS: Platform as a Service
PAT: Autonomous Province of Trento
TS4.0: TrentinoSalute4.0
USCA: Unità Speciali di Continuità Assistenziale
