# Supplementary figures and images for: Use of eHealth Platforms and Apps to Support Monitoring and Management of Home-Quarantined Patients With COVID-19 in the Province of Trento, Italy: App Development and Implementation
Source: JMIR Form Res. 2021 May 31;5(5):e25713. doi: 10.2196/25713 (PMC8168637; doi:10.2196/25713)

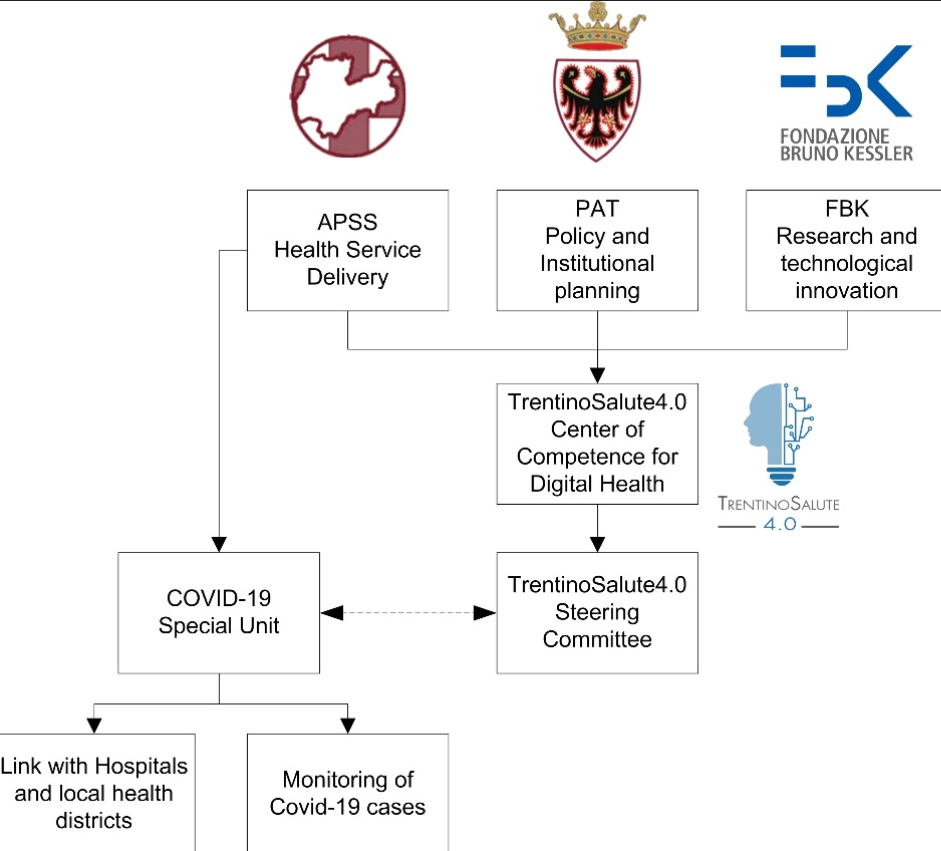

Supplement: Multimedia Appendix 1 [file formative_v5i5e25713_app1.png]
