# Supplementary material for: Use of eHealth Platforms and Apps to Support Monitoring and Management of Home-Quarantined Patients With COVID-19 in the Province of Trento, Italy: App Development and Implementation
Source: JMIR Form Res. 2021 May 31;5(5):e25713. doi: 10.2196/25713 (PMC8168637; doi:10.2196/25713)
Supplement: Multimedia Appendix 2 [file formative_v5i5e25713_app2.docx]

**Supplementary Table: Detailed questionnaire (Italian version with English translation).**

Table 1 of the manuscript details the key pieces of information that have been identified by the team of medical doctors and nurses in charge of monitoring the disease progression, and that was used to develop the automated alarming system. The detailed questionnaire used through the App is provided here as supplementary material. The questionnaire was designed and used in Italian. In the following table an English translation is provided.

| **Item number** | **Question (original version in Italian)** | **Question (English translation)** |
| --- | --- | --- |
| 1 | Se sei in grado di rispondere come ti senti in questo momento?  Vigile; Confuso-disorientato; Non risvegliabile | If you are able to answer how do you feel right now?  Alert and oriented; Confused-disoriented; Not awakened |
| 2 | Qual è il valore della tua temperatura corporea (febbre)? (>34, <44) | What is your body temperature (fever)? (> 34, <44 C) |
| 3 | Assumi farmaci per la febbre? (SI/NO) | Are you taking fever medications? (YES / NO) |
| 4 | Mangi e bevi come al solito? (SI/NO) | Do you eat and drink as usual? (YES / NO) |
| 5 | Quanto ti senti stanco? Inserisci un valore da zero (per nulla) a 10 (moltissimo) (0–10) | How tired do you get? Enter a value from zero (not at all) to 10 (a lot) (0-10) |
| 6 | Hai la tosse? Se sì indica se è la prima volta o come si presenta rispetto a ieri?  a) Non ho la tosse  b) Sì è la prima volta  c) Sì è migliorata  d) Sì è stazionaria  e) Sì è peggiorata | Do you have cough? If so, indicate if it is the first time or how it looks compared to the cough you had yesterday?  a) I don't have cough  b) Yes, it is the first time  c) Yes, it has improved  d) Yes, it is stationary/stable  e) Yes, it got worse |
| 7 | Qual è il grado da zero (per nulla) a dieci (moltissimo) di difficoltà respiratoria che percepisci? (0–10) | From zero (not at all) to ten (very much), what is the level of breathing difficulty that you have? (0-10) |
| 8 | Se hai il saturimetro indica il valore percentuale di saturazione (SpO2) misurato attraverso il saturimetro, altrimenti inserisci zero. (0 - 100%) | If you have an oximeter with you, please indicate the percentage/value of saturation (SpO2) measured through the oximeter; otherwise enter zero. (0 - 100%) |
| 9 | Se hai il saturimetro inserisci il numero di battiti cardiaci rilevati, altrimenti inserisci zero | If you have an oximeter with you, please indicate the number of heartbeats detected; otherwise enter zero |
| 10 | Qual è la frequenza respiratoria? Indica il numero di respiri che fai al minuto (per sapere come fare questa misurazione vai nella sezione “Video Tutorial” della Home e guarda il video “l’autocontrollo della frequenza respiratoria”. La persona che ti assiste può guardare il video “il controllo della frequenza respiratoria”) (0-100) | What is the respiratory rate? Please indicate the number of breaths you take per minute (to find out how to do this measurement go to the "Video Tutorial" section of the Home and watch the video "self-monitoring of the respiratory rate". The person/caregiver assisting you can also watch the video "respiratory rate ") (0-100) |
| 11 | A livello del polpaccio presenti dolore, gonfiore, calore o rossore? Rispondi sì in presenza di uno o più dei disturbi elencati (SI/NO) | Do you have pain, swelling, warmth or redness in your calf? Answer yes in case of one or more of the following symptoms (YES / NO) |
| 12 | E per finire se hai misurato la pressione arteriosa, inserisci il valore della pressione minima (diastolica) (0-300). E ora il valore della pressione massima (sistolica) (0-300) | And finally, if you have measured your blood pressure, enter the value of the minimum (diastolic) pressure (0-300). And now the value of the maximum (systolic) pressure (0-300) |
| 13 | Confermi che ad oggi hai rispettato le norme di isolamento? (SI/NO) | Do you confirm that today you have followed the isolation regulations? (YES / NO) |
| 14 | Confermi i seguenti valori? Riepilogo dei valori (SI/NO) - Se non corrispondono vengono riproposti per la correzione | Do you confirm the following values? Summary of values ​​(YES / NO). Patient is in the position of amending/correcting the values |
|  |  |  |
| *Message* | *I tuoi dati sono stati inviati correttamente. Ti ricordo di inserire le prossime rilevazioni: una alla mattina tra le 10 e le 12 e una al pomeriggio tra le 16 e le 18. Grazie per il tempo dedicato, buona giornata e a presto.* | *Your data has been sent successfully. Let me remind you to enter the next measurements according to the agreed timeline: once in the morning between 10 and 12, and once in the afternoon between 16 and 18. Thank you and have a good day.* |
|  |  |  |
| *Note* | *Sta all’operatore decidere se aumentare la frequenza di inserimento dati. Nel caso di 3 inserimenti giornalieri:*   - *8.30 / 10.30* - *12.30 / 14.30* - *16 / 18* | *It is up to the healthcare staff to decide whether to increase the data entry frequency. In case of 3 daily entries, the slots are as follows:*   - *8.30 / 10.30* - *12.30 / 14.30* - *16 / 18* |
